# Elevated Ferritin Levels Associated with High Body Fat Mass Affect Mortality in Peritoneal Dialysis Patients

**DOI:** 10.3390/nu15092149

**Published:** 2023-04-29

**Authors:** Hyung Seok Lee, Hye-Mi Noh, Jung Nam An, Young Rim Song, Sung Gyun Kim, Jwa-Kyung Kim

**Affiliations:** 1 Department of Internal Medicine & Kidney Research Institute, Hallym University Sacred Heart Hospital, Anyang 14068, Republic of Korea; 2 Department of Family Medicine, Hallym University Sacred Heart Hospital, Anyang 14068, Republic of Korea

**Keywords:** peritoneal dialysis, obesity, inflammation, ferritin, mortality

## Abstract

Obesity is a common health problem in peritoneal dialysis (PD) patients and causes high serum ferritin levels. However, mixed results have been reported on whether serum ferritin levels affect the prognosis of PD patients. We investigated the effect of increased adiposity on ferritin levels and its association with mortality in 350 well-nourished PD patients. Body composition was measured using a portable whole-body bioimpedance spectroscope, and clinical determinants of high ferritin levels were evaluated. High ferritin levels (≥600 ng/mL) were observed in 63 (18.0%) patients. Patients with high ferritin levels had a significantly higher body fat percentage and a lower lean tissue index than patients with low or normal ferritin levels. During a median follow-up of 30 months, there were 65 deaths. Ferritin ≥ 600 ng/mL was associated with significantly higher all-cause mortality compared with 200–600 ng/mL of ferritin. Multivariate analysis showed that high ferritin levels were significantly associated with a higher percentage of body fat after adjustment for lean tissue index and volume status. High ferritin increased all-cause mortality in PD patients, and increased fat mass was an important determinant of the high ferritin. Our results support that adiposity may lead to an adverse clinical outcome in PD patients.

## 1. Introduction

Peritoneal dialysis (PD) rates have been declining worldwide, but its advantage as a home-based therapy has recently gained traction [1,2]. In addition, the long-term survival rate of PD patients has improved with advances in the connection system and standardization of the treatment protocol [3]. Thus, the care of PD patients has shifted to improving longevity and quality of life and minimizing metabolic complications [4,5,6].

Obesity is a common treatment-related complication of PD [7]. A large proportion of patients have experienced significant weight gain during PD treatment [8,9]. Unfortunately, although the obesity paradox has been well-documented in patients with advanced chronic kidney disease (CKD) or on dialysis, the available literature does not show a consistent prognostic effect of weight gain or obesity in PD patients [10,11,12]. In addition, body mass index (BMI), which is used to define obesity, has a limited role in predicting prognosis because it does not distinguish between lean and adipose tissue mass or overhydration status [13]. Incident PD patients with a high BMI may have better survival rates if they have high muscle mass [14]. However, if the high BMI is accompanied by increased adiposity with frailty, this may be associated with poor outcomes [13,15]. With previous data showing that increased serum leptin levels during PD are associated with inflammation and a decrease in lean body mass, becoming obese during PD may have long-term adverse effects [16].

One of the possible mechanisms by which increased adiposity adversely affects prognosis is its association with iron metabolism. Abdominal fat is a source of inflammatory cytokines; therefore, increased adiposity causes systemic inflammation, which increases serum ferritin levels and induces overproduction of hepcidin [17]. High hepcidin levels slow down the release of recycled iron from iron stores by macrophages and decrease intestinal iron absorption [18,19]. As a result, CKD patients with high adiposity may develop abnormal iron metabolism, hyperferritinemia, and a functional iron deficiency [20]. Ferritin is an iron storage marker that, along with transferrin saturation (TSAT), is commonly used to guide iron use in CKD patients [21]. However, chronic low-grade inflammation also determines the ferritin concentration. Unlike hemodialysis (HD) patients, the use of intravenous iron in PD patients is limited. Therefore, high ferritin levels in PD patients are rarely caused by an overdose of parenteral iron, but rather reflect systemic inflammation, especially in obese patients [16]. To date, mixed results have been reported on whether high serum ferritin levels affect the prognosis of dialysis patients [22,23]. However, hyperferritinemia has been documented as a prognostic factor for mortality in other obesity-related chronic inflammatory conditions, such as type 2 diabetes mellitus, metabolic syndrome, and non-alcoholic fatty liver disease [24,25].

To date, it is unclear whether hyperferritinemia can be a predictive biomarker of poor prognosis in PD patients, independent of anemia or inflammation. In addition, the effect of increased adiposity on ferritin levels has not been investigated in this population. Therefore, the present study evaluated the prognostic role of high ferritin levels in prevalent PD patients and determined the role of high body fat mass in plasma ferritin concentrations.

## 2. Materials and Methods

### 2.1. Study Design and Population

We gathered our prevalent PD cohort of patients between January 2013 and December 2020. At our center, all PD patients are regularly followed-up with monthly, and the peritoneal equilibration test (PET) and PD adequacy are measured every 6–12 months. In addition, we perform a bioimpedance test twice a year. Using a portable whole-body bioimpedance spectroscopy device (Body Composition Monitor [BCM], Fresenius Medical Care, Bad Homburg, Germany), we obtained objective indicators of fluid status, including estimates of overhydration (OH), extracellular water (ECW), intracellular water, total body water (TBW), fat tissue index (FTI), percentage of body fat (PBF, %), and lean tissue index (LTI). Overhydrated status was defined as OH > 2.5 L in each patient. This study was approved by the Institutional Review Board/Ethics Committee of Hallym University Sacred Heart Hospital, Anyang, Korea. The IRB waived the requirement for informed consent because this was a retrospective analysis of collected data.

Exclusion criteria were age < 20 years, a recent history of infection, hospitalization < 3 months, severe malnutrition (serum albumin < 2.5 g/dL), and refusal of BCM. We used only the first bioimpedance test of each patient for those who repeated the test. During the study period, 365 prevalent patients were enrolled, and 15 cases were excluded for the following reasons: transfer to another center (*n* = 5), reaching the endpoint within 3 months after the BCM test (*n* = 6), and inadequate BCM test (*n* = 4). In the end, 350 patients were analyzed. Patients were censored at a modality change (transition to HD or kidney transplant) or transferred to another center.

### 2.2. Covariates

Data collected included age, sex, the underlying cause of renal disease (diabetic kidney disease, hypertensive nephrosclerosis, glomerulonephritis, and others), comorbidities (history of coronary artery disease, cerebrovascular accident), duration of PD, and BMI. Baseline BMI was calculated as body weight/(height)^2^. Obesity was defined as a baseline BMI ≥ 25 kg/m^2^. Serum levels of albumin, cholesterol, creatinine, and normalized protein nitrogen appearance (nPNA) were measured to estimate nutritional status. In addition, high-sensitivity C-reactive protein (hs-CRP) levels were measured. Iron profile, serum iron levels, total iron binding capacity (TIBC), and ferritin levels were measured, and TSAT was calculated as iron/TIBC × 100. Increased adiposity was defined as a higher PBF than the sex-specific median value in our cohort (>25.0% for men and >33.0% for women). Lower LTI was defined as the sex-specific median value (<15.6% for men and <13.2% for women).

### 2.3. Endpoint

The study endpoint was all-cause mortality. Because this study was based on a well-managed PD cohort, the cause of death was determined. Sudden cardiac death was considered CV mortality.

### 2.4. Statistical Analysis

Statistical analyses were performed with SPSS software (ver. 24.0; SPSS Inc., Chicago, IL, USA). All variables are expressed as the mean ± standard deviation or as median and range. Patients were divided into three groups according to baseline ferritin levels (<200, 200–599, ≥600), and the differences between the groups were determined using an independent *t*-test and analysis of variance for continuous variables or the χ^2^ test for categorical data. The effect of high ferritin levels on all-cause mortality and CV mortality was assessed using a Cox proportional hazards model. All models were adjusted for multiple covariates (age, sex, diabetes, hemoglobin, CRP, and OH). Pearson’s correlation analysis was used to clarify the relationships between ferritin, TSAT, and parameters including adiposity, nutritional markers, and CRP. Multiple logistic regression analysis was performed to evaluate the determinants of high ferritin levels (>600 ng/mL). The predictive value of higher PBF for high ferritin was expressed as the odds ratio (OR) with corresponding 95% confidence intervals (CIs). We performed causal mediation analysis to investigate the effect of PBF (independent variable) on mortality (dependent variable) via ferritin (third variable, called a mediator). The bootstrap procedure was used to determine the significance of the mediated effects. We determined 95% Cis from the 5000 bootstrap resamples, and any interval that did not include 0 was considered to be statistically significant. All analyses were performed using IBM SPSS Statistics for Windows version 28.0 (IBM Corp., Armonk, NY, USA) and R version 4.1.0. A *p*-value < 0.05 was considered significant.

## 3. Results

### 3.1. Baseline Characteristics

The study population consisted of 350 patients over 8 years. The mean age was 54.7 years; 67.1% (*n* = 235) were men and 54.6% (*n* = 191) had diabetes. The median dialysis duration was 22 months (interquartile range, 7.4–51.4 months). More than half of the patients were obese (mean BMI, 25.4 kg/m^2^), and severe obesity was observed in 39 patients (11.1%). The mean serum ferritin level was 381.1 ± 316.0 ng/mL; the numbers of patients with ferritin levels <200, 200–599, and ≥600 ng/mL were 99 (28.3%), 188 (53.7%), and 63 (18.0%), respectively. Baseline characteristics among the three ferritin groups are compared in Table 1. Patients with high ferritin levels (≥600 ng/mL) were significantly older (*p* = 0.014), were more likely to be male (*p* < 0.001), tended to have longer dialysis duration (*p* < 0.001), and more commonly had a history of coronary artery disease (CAD) (*p* = 0.009). In addition, white blood cells (*p* < 0.001) and serum inflammatory markers, CRP (*p* < 0.001), were significantly higher in the high ferritin group, but hemoglobin (*p* < 0.001), TIBC (*p* < 0.001), and nPNA levels (*p* = 0.014) were significantly lower in this group. Importantly, among the patients with ferritin ≥ 600 ng/mL, the mean hemoglobin level was 9.6 ± 1.1 g/dL, and almost 70% of the patients were anemic, with hemoglobin < 10.0 g/dL, although the mean TSAT was 43.5 ± 23.0%. However, there were no differences in BMI, albumin, or cholesterol among the three groups.

**Figure 1:**
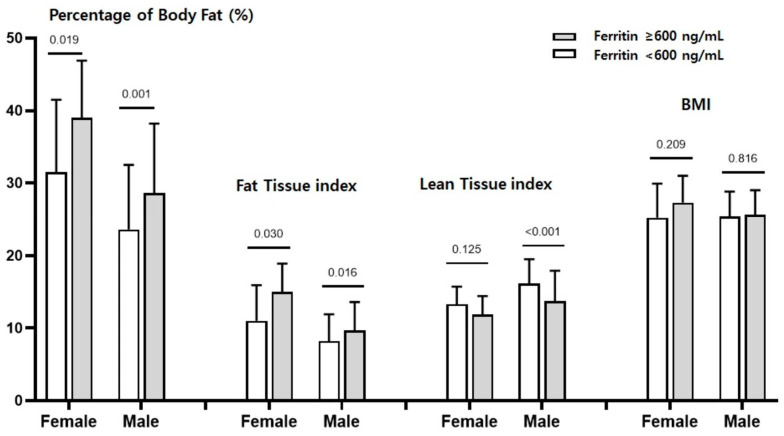
Comparisons of gender-specific body fat percentage, fat tissue index, lean tissue index, and BMI by ferritin levels ≥ 600 ng/mL and <600 ng/mL. Patients in the high ferritin group had significantly more fat than those in the low ferritin group. Differences in lean tissue index were observed only in male patients. BMI did not differ by ferritin level.

In body composition analysis, median LTI, FTI, and PBF were, respectively, 15.6 ± 3.5 kg/m^2^, 8.5 ± 3.8 kg/m^2^, and 24.8 ± 9.3% in men, and 13.1 ± 2.4 kg/m^2^, 11.5 ± 5.0 kg/m^2^, and 33.2 ± 10.1% in women. Surprisingly, PBF, FTI, and LTI differed significantly by ferritin level: patients with high ferritin levels (≥600 ng/mL) had significantly lower LTI but higher FTI and PBF (Figure 1). In addition, as shown in Table 1, the marker of volume status, the ECW/TBW ratio, was significantly higher in the high ferritin group, but no differences in the OH or serum Na levels were found among the three groups.

### 3.2. Increased Adiposity and High Ferritin Levels

Correlation analyses showed that ferritin correlated with age (r = 0.140, *p* = 0.009), dialysis duration (r = 0.243, *p* < 0.001), WBC count (r = 0.167, *p* = 0.002), hemoglobin (r = −0.200, *p* < 0.001), and CRP (r = 0.298, *p* < 0.001). In addition, ferritin showed a significant association with low LTI (r = −0.154, *p* = 0.004), high ECW/TBW ratio (r = 0.169, *p* = 0.002), and high PBF (r = −0.135, *p* = 0.011) (Table 2). However, TSAT (%) had no association with any of the laboratory or demographic parameters, except for iron or TIBC levels. Notably, serum albumin was not correlated with ferritin levels in our PD patients.

### 3.3. High Ferritin Levels and Their Impact on All-Cause Mortality

**Figure 2:**
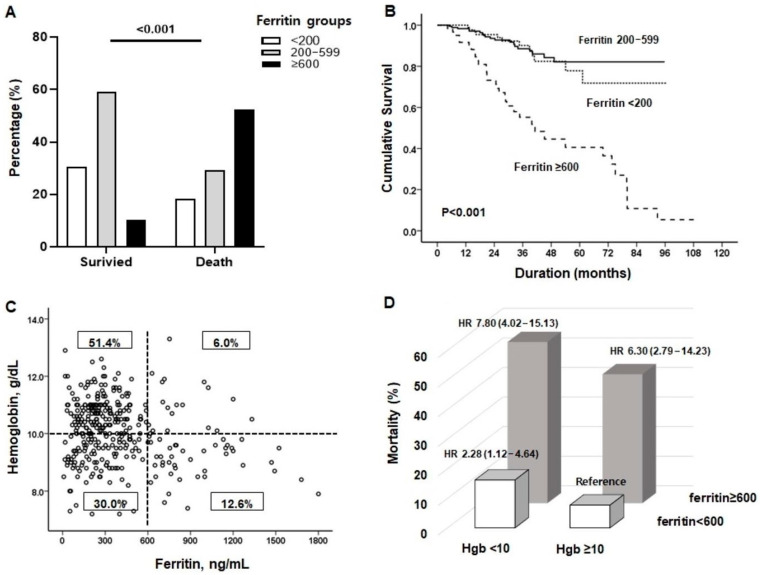
(**A**,**B**) Mortality differed significantly among three ferritin groups (<200, 200–599, ≥600 ng/mL). Patients in the high ferritin group had the worst survival rate among PD patients. (**C**) To determine the prognostic role of high ferritin levels independent of anemia, the patients were divided into four groups according to ferritin levels of 600 ng/mL and hemoglobin levels of 10.0 g/dL. (**D**) High ferritin levels increased mortality rates regardless of hemoglobin levels, but the highest mortality rates were observed in patients with high ferritin combined with low hemoglobin.

During a median follow-up of 30 months (interquartile range: 18–45 months), 65 deaths (18.6%) occurred, resulting in a mortality rate of 60.3 per 1000 patient-years. The most common causes of death were CV events (*n* = 36), infections (*n* = 25), and other (*n* = 4). Table 3 shows the risk factors for mortality in prevalent PD patients. As expected, univariate analysis showed that well-known risk factors, such as old age, the presence of diabetes, longer dialysis duration, anemia, high CRP, and OH > 2.5 L significantly increased the risk of death. Among the three ferritin groups, the mortality rate was lowest in patients with ferritin levels of 200–599 ng/mL, and a high ferritin level ≥ 600 ng/mL increased the risk of death by 5.5-fold. Kaplan–Meier survival analysis also revealed a high mortality rate with ferritin ≥ 600 ng/mL (Figure 2A,B). Surprisingly, among the 65 patients who died, more than half (*n* = 34, 52.3%) had ferritin levels ≥ 600 ng/mL, but we found no association between this and the causes of death. Multivariate analysis adjusting for all of the risk factors showed that age ≥ 65 years, longer dialysis duration, overhydration (OH > 2.5 L), and ferritin ≥ 600 ng/mL were significant risk factors for high mortality (Table 3).

In addition, we divided the population into four groups according to a ferritin level of 600 ng/mL and hemoglobin of 10.0 g/dL to determine the prognostic role of high ferritin levels on mortality independent of anemia (Figure 2C). As shown in Figure 2D, a high ferritin level itself was associated with a much higher risk of death, but the mortality rate was the highest when combined with anemia (12.6% of patients) (HR 7.80, CI 4.02–15.13).

### 3.4. The Effect of High Fat Mass on Ferritin Levels

Next, we identified the clinical factors influencing high ferritin levels ≥ 600 ng/mL in PD patients. As shown in Table 4, univariate analyses showed that older age, male sex, longer dialysis duration, high CRP level, high-fat percentage, and low muscle mass affected high ferritin levels ≥ 600 ng/mL. Interestingly, BMI and OH were not significant. Even after adjusting for these parameters, high-fat percentage and high CRP levels were significant determinants of high ferritin levels in prevalent PD patients (Table 4).

### 3.5. Causal Mediation

**Figure 3:**
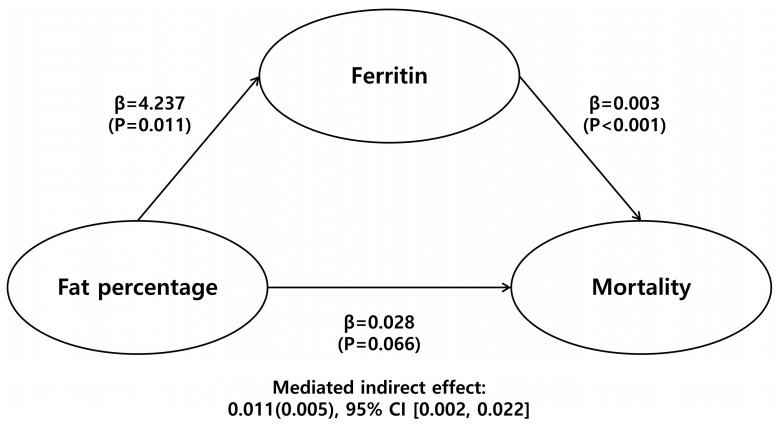
Mediation analysis results are β (*p*-value), indirect effect is β (standard error), 95% confidence interval.

The results of the mediation analysis showed that the association between PBF and mortality is mediated by ferritin levels. The indirect effect between PBF and mortality through ferritin levels was significant (β = 0.011 (standard error: 0.005), bootstrapped 95% CI [0.002, 0.022]). However, the direct effect of the PBF on mortality was not significant (β = 0.028 (standard error: 0.015), bootstrapped 95% CI [−0.002, 0.058]) (Figure 3).

## 4. Discussion

In this study, we found that high-fat mass in PD patients was closely associated with significantly high serum ferritin levels, independent of age, volume status, or inflammatory status. The high ferritin level, in turn, was associated with high mortality and a poor prognosis in these patients. The long-term prognostic effect of a high ferritin level in prevalent PD patients, as well as its association with increased adiposity, is novel, and our data suggest that it is important to minimize excess fat gain in prevalent PD patients. In addition, our results showed a strong association between high ferritin, inflammation, and anemia, which may contribute to the high ferritin-related increased mortality in PD patients. Mediation analysis revealed that body fat did not have a significant direct effect on mortality, but it only had a significant indirect effect through ferritin.

Obesity is becoming increasingly common in PD patients. Previous data have shown that significant weight gain occurs primarily during the early period after initiation of PD and that this weight gain is often accompanied by increased fat mass and central obesity [26]. Peritoneal glucose overload, old age, diabetes, and genetic polymorphisms, such as the uncoupling protein 2 exon 8 insertion/deletion, are known as risk factors for weight gain [27,28]. In addition, some data have shown that improvement in uremia and general health with PD therapy is a more important cause of weight gain than glucose absorption from the dialysate [29]. Although the clinical significance of obesity in this population remains unclear, a recent long-term study of 950 incident PD patients showed that weight gain > 3 kg was associated with worse survival regardless of the baseline BMI, suggesting that weight gain may not be beneficial in PD patients. However, other studies on the relationship between obesity and the outcome in PD patients have shown conflicting results [12,13,30]. The reason may be that BMI is not an accurate marker of adiposity and, indeed, BMI could not differentiate body composition [31]. Incident PD patients with a high BMI may survive longer if they have high skeletal muscle mass rather than high adipose tissue mass. However, a high BMI with high-fat mass may not be associated with better outcomes, as shown by the J-curve phenomenon [15]. Supporting this, a Chinese study of 267 prevalent PD patients reported that central adiposity, or a high waist-to-hip ratio, was strongly associated with frailty and poor outcome [32]. Indeed, obesity has emerged as a key modifiable risk factor bridging the traditional and non-traditional pathogenic pathways of CV disease.

Serum ferritin levels serve as a marker of iron stores, along with TSAT, which is commonly used to guide intravenous iron dosing practices in dialysis patients. However, ferritin increases when patients are inflamed; therefore, it correlates more with markers of acute illness than with iron stores [22,33,34]. Kalantar-Zadeh et al. showed that high ferritin levels were strongly associated with high mortality in HD patients [35]. Similarly, a recent Taiwanese study of 4356 well-nourished PD patients showed that serum ferritin levels > 800 ng/mL were associated with a higher risk of all-cause mortality [36]. In our data, ferritin ≥ 600 ng/mL increased all-cause mortality by 3.85-fold. Our finding is noteworthy because we adjusted for many known risk factors to assess mortality risk, such as age, anemia, diabetes, history of CAD, high CRP level, and volume overload status, over a long duration. Importantly, high ferritin levels contributed to mortality independently of anemia or iron status. In fact, the high ferritin group at baseline had a significantly higher TSAT at baseline than the low or normal ferritin groups, even though the hemoglobin level was significantly lower. Of course, the opposite result has also been reported in PD patients. In 3734 Japanese PD patients, there was no clear association between serum ferritin levels and mortality in PD patients [37], but, because the follow-up period in this study was only 1 year, the effect of ferritin levels on long-term mortality is unknown. In fact, in our data, Kaplan–Meier analysis did not show a significant difference for one year.

In the correlation analysis, we found a significant correlation between high-fat mass, elevated CRP levels, and ferritin levels. Additionally, ferritin correlated with the ECW/TBW ratio but not with OH. To explain this finding, we evaluated the correlation between an inflammatory marker (i.e., CRP) and the ECW/TBW or OH ratio. Interestingly, CRP level was positively associated with ECW/TBW but not with OH, suggesting that the close relationship between ferritin and the ECW/TBW ratio may be due to their close association with an inflammatory state. Notably, however, neither albumin level nor BMI was correlated with ferritin level. In general, patients with high ferritin levels and chronic inflammation are expected to have low serum albumin levels, and hypoalbuminemia is considered a negative prognostic factor in many diseases. However, in our data, serum albumin and BMI levels were similar among the three ferritin groups. We believe this is because most of our patients were well nourished. The interquartile range of serum albumin and BMI were 3.3–3.8 g/dL and 23.0–27.8 kg/m^2^, respectively, but nPNA level was significantly lower in the high ferritin group than in the others.

Another important finding of this study is that high-fat mass (i.e., increased adiposity) was an important determinant of high ferritin levels in prevalent PD patients. A body fat percentage higher than the median is a significant predictor of high ferritin levels. Multivariate analyses showed that high CRP and high-fat percentage were major contributors to high ferritin levels, even after adjusting for low muscle mass. Our data suggest that increased adiposity in PD patients may adversely affect mortality by increasing ferritin levels. Supporting our findings, high adiposity is characterized by chronic low-grade inflammation, increased hepcidin, and decreased iron absorption, eventually leading to anemia and high ferritin levels [38]. Hyperferritinemia has been documented in obesity-related chronic inflammatory conditions, such as type 2 diabetes mellitus, metabolic syndrome, and nonalcoholic fatty liver disease [24]. However, to the best of our knowledge, our study is the first to demonstrate the role of high fat mass in increasing serum ferritin levels in PD patients.

This study had several limitations. First, as a retrospective analysis of prospectively-collected data, we could not determine the percentage of parenteral iron used in our cohort. However, compared with HD, the recommendations for intravenous iron supplementation are still unknown and, in practice, most PD patients do not use intravenous iron frequently. In addition, evidence of iron supplementation on outcomes in PD patient has rarely been reported. Similarly, the types and doses of erythropoiesis-stimulating agents (ESAs) varied among the patients, and we could not calculate the mean dose of ESAs, so the effects of ESAs on the ferritin levels could not be considered. Second, this was a single-center study with a relatively small number of patients. Therefore, our results do not represent the entire PD population. However, with the well-characterized PD cohort, we obtained detailed data on biochemical profiles, body composition analysis, and long-term outcomes in this study. Finally, although we measured serum CRP levels, the level was not quantified if it was <1.0 mg/L. Therefore, we semi-quantitatively divided the CRP levels into <1.0 or ≥1.0 mg/L groups. This analysis seemed quite reasonable, as it correlated well with other inflammatory parameters, such as WBC and ferritin levels.

## 5. Conclusions

In conclusion, a high ferritin level (≥600 ng/mL) was associated with a higher risk of all-cause mortality in PD patients, and its effects were independent of age, anemia, and volume status. High adiposity with a high-fat mass was a predictor of high ferritin levels. Therefore, we recommend avoiding excessive fat gain in prevalent PD patients.

## Figures and Tables

**Table 1:** Baseline characteristics of study subjects.

Variables		Ferritin			*p*
	Total (*n* = 350)	<200 (*n* = 99, 28.3%)	200–599 (*n* = 188, 53.7%)	≥600 (*n* = 63, 18.0%)	
Age (years)	54.7 ± 11.1	54.0 ± 11.1	53.9 ± 11.4	58.4 ± 10.2	0.014
≥65 years, *n* (%)	72 (20.6)	15 (15.2)	37 (19.7)	20 (31.7)	0.042
Gender, male, *n* (%)	235 (67.1)	52 (52.5)	129 (68.6)	54 (85.7)	<0.001
Dialysis duration	36.0 ± 28.8	24.4 ± 15.5	37.8 ± 30.4	48.5 ± 40.2	<0.001
Diabetes, *n* (%)	191 (54.6)	54 (54.5)	96 (51.1)	41 (65.1)	0.150
Coronary artery disease	36 (10.3)	5 (5.1)	18 (9.6)	13 (20.5)	0.009
SBP, mmHg	144.1 ± 22.2	150.5 ± 21.2	142.4 ± 23.3	139.2 ± 22.3	0.003
DBP, mmHg	83.0 ± 13.4	84.3 ± 11.9	82.7 ± 14.2	79.2 ± 13.3	0.144
BMI, kg/m^2^	25.4 ± 4.2	25.8 ± 4.2	25.0 ± 4.3	25.5 ± 3.9	0.635
≥25.0, *n* (%)	183 (52.3)	50 (50.5)	97 (51.6)	36 (57.1)	0.684
nPNA, g/kg	0.75 ± 0.37	0.70 ± 0.37	0.83 ± 0.37	0.62 ± 0.34	0.014
WBC, ×10^3^/μL	6.7 ± 1.7	6.7 ± 1.4	6.4 ± 1.6	7.5 ± 2.0	<0.001
Hemoglobin, g/dL	10.1 ± 1.0	10.1 ± 1.0	10.3 ± 1.0	9.6 ± 1.1	<0.001
<9.0	58 (16.6)	14 (14.1)	23 (12.2)	21 (33.3)	0.001
<10.0	149 (42.6)	41 (41.4)	64 (34.0)	44 (69.8)	0.001
Ferritin, ng/mL	381.1 ± 316.0	114.9 ± 53.0	339.2 ± 102.2	932 ± 314	<0.001
Iron, μg/dL	74.5 ± 35.6	70.9 ± 34.9	73.7 ± 31.3	83.5 ± 42.5	0.027
TIBC, μg/dL	231.7 ± 54.7	263.5 ± 49.7	225.2 ± 49.6	199.5 ± 53.3	<0.001
TSAT, %	33.2 ± 16.4	27.3 ± 13.3	32.9 ± 13.5	43.5 ± 23.0	<0.001
<20%, *n* (%)	67 (19.3)	30 (30.3)	30 (16.7)	7 (11.3)	0.003
BUN, mg/dL	68.5 ± 21.9	63.3 ± 20.4	72.8 ± 21.8	63.2 ± 22.0	0.967
Creatinine, mg/dL	12.5 ± 6.8	11.4 ± 4.4	13.4 ± 8.1	11.5 ± 4.7	0.887
Serum calcium, mg/dL	8.5 ± 0.9	8.3 ± 1.0	8.5 ± 0.9	8.7 ± 0.8	0.067
Serum phosphate, mg/dL	5.6 ± 1.5	5.7 ± 1.6	5.7 ± 1.6	5.2 ± 1.7	0.183
Total cholesterol, mg/dL	155.8 ± 37.8	152.2 ± 43.4	159.0 ± 34.1	151.6 ± 38.0	0.760
Albumin, g/dL	3.5 ± 0.4	3.5 ± 0.3	3.5 ± 0.5	3.5 ± 0.4	0.707
Na, mmol/L	135.7 ± 4.3	134.8 ± 4.5	136.0 ± 4.0	135.5 ± 4.9	0.134
C-reactive protein, ≥1.0 mg/L	170 (48.5)	41 (41.4)	75 (39.8)	54 (85.7)	<0.001
Volume status					
OH, L	1.8 ± 2.1	1.8 ± 1.8	1.7 ± 2.1	2.2 ± 2.3	0.136
ECW/TBW ratio	0.47 ± 0.03	0.47 ± 0.03	0.46 ± 0.03	0.49 ± 0.04	0.060
Oral iron, *n* (%)	189 (54.0)	83 (83.8)	101 (53.7)	5 (7.9)	0.001

All data are expressed as mean ± standard deviation or frequencies (percentage), as appropriate. SBP, systolic blood pressure; DBP, diastolic blood pressure; BMI, body mass index; nPNA, normalized protein nitrogen appearance; TIBC, total iron binding capacity; TSAT, transferrin saturation; OH, overhydration; ECW, extracellular water; TBW, total body water.

**Table 2:** Correlation analysis between ferritin levels and clinical markers in PD patients.

	Ferritin	
	Correlation Coefficient	*p*
Age	0.140	0.009
SBP	−0.168	0.002
BMI	0.018	0.740
Dialysis vintage	0.243	<0.001
WBC	0.167	0.002
Hemoglobin	−0.200	<0.001
Iron	0.132	0.014
TIBC	−0.366	<0.001
CRP ≥ 1.0	0.298	<0.001
TSAT (%)	0.342	<0.001
Albumin	0.008	0.879
OH	0.041	0.448
ECW/TBW	0.169	0.002
LTI	−0.154	0.004
Fat percentage (%)	0.136	0.011

Abbreviations: SBP, systolic blood pressure; BMI, body mass index; TIBC, total iron binding capacity; TSAT, transferrin saturation; OH, overhydration; ECW, extracellular water; TBW, total body water; LTI, lean tissue index.

**Table 3:** The risk of all-cause mortality.

	Univariate		Multivariate *		Multivariate ^†^	
	HR (95% CI)	*p*	HR (95% CI)	*p*	HR (95% CI)	*p*
Age ≥ 65	5.28 (3.22–8.66)	<0.001	4.67 (2.80–7.79)	<0.001	4.37 (2.39–7.99)	<0.001
Male	2.0 (1.10–3.60)	0.022	1.41 (0.34–1.80)	0.217	1.25 (0.45–1.49)	0.315
Diabetes	2.54 (1.47–4.39)	<0.001	2.13 (1.21–3.72)	0.008	1.78 (0.87–3.62)	0.113
PD duration, month	1.01 (1.00–1.013)	0.040	1.01 (1.00–1.013)	0.054	1.01 (1.002–1.018)	0.011
Ferritin						
200–600	1 (Reference)		1 (Reference)		1 (Reference)	
<200	1.16 (0.56–2.39)	0.682	1.87 (0.85–4.06)	0.116	1.71 (0.72–4.06)	0.220
≥600	5.53 (3.15–9.74)	<0.001	4.01 (2.26–7.11)	<0.001	4.58 (2.05–10.23)	<0.001
Hemoglobin < 10.0	2.52 (1.52–4.18)	0.002	-	-	0.89 (0.43–2.76)	0.728
Albumin < 3.0	1.13 (0.68–1.87)	0.628	-	-		
CRP ≥ 1.0	3.32 (1.76–6.25)	<0.001	-	-	1.45 (0.70–3.00)	0.313
OH > 2.5 L	2.00 (1.24–3.33)	0.008	-	-	2.02 (1.04–3.93)	0.037

* Adjusted for age ≥ 65, sex, DM, PD duration, and ferritin group. ^†^ Adjusted for age ≥ 65, sex, DM, PD duration, ferritin group, hemoglobin < 10.0 g/dL, CRP ≥ 1.0, and OH > 2.5 L.

**Table 4:** Multivariate analysis for clinical factors affecting high ferritin levels ≥ 600 ng/mL in PD patients.

	Univariate Analysis		Multivariate Analysis *	
	OR (95% CI)	*p*	OR (95% CI)	*p*
Age ≥ 65	2.10 (1.14–3.86)	0.017	1.60 (0.72–3.57)	0.249
Male	3.51 (1.66–7.40)	0.001	3.21 (1.29–8.08)	0.013
Diabetes	1.70 (0.97–3.00)	0.066	-	-
Dialysis duration > 3 years	1.84 (1.05–3.20)	0.032	1.53 (0.74–3.15)	0.248
CRP ≥ 1.0	8.80 (3.80–20.34)	<0.001	6.03 (2.53–14.40)	<0.001
OH > 2.5 L	1.30 (0.74–2.29)	0.357	-	-
PBF (%) > median	3.36 (1.84–6.14)	<0.001	3.22 (1.31–7.94)	0.011
LTI < median	2.89 (1.60–5.25)	<0.001	1.57 (0.65–3.81)	0.315
BMI ≥ 25	1.27 (0.73–2.20)	0.395	-	-

Abbreviations: OH, overhydration; PBF, percentage of body fat; LTI, lean tissue index; BMI, body mass index. * Adjusted for age ≥ 65, sex, dialysis duration, CRP levels, PBF (%), and LTI.

## Data Availability

The data presented in this study are available on request from the corresponding author.
